# Long-Term Encapsulated Nitrate Supplementation Modulates Rumen Microbial Diversity and Rumen Fermentation to Reduce Methane Emission in Grazing Steers

**DOI:** 10.3389/fmicb.2019.00614

**Published:** 2019-03-29

**Authors:** Yury Tatiana Granja-Salcedo, Rodolfo Maciel Fernandes, Rafael Canonenco de Araujo, Luciano Takeshi Kishi, Telma Teresinha Berchielli, Flávio Dutra de Resende, Alexandre Berndt, Gustavo Rezende Siqueira

**Affiliations:** ^1^Department of Animal Science, Faculdade de Ciências Agrárias e Veterinárias, UNESP – Universidade Estadual Paulista, Jaboticabal, Brazil; ^2^Department of Animal Science, Agência Paulista de Tecnologia dos Agronegócios, Colina, Brazil; ^3^GRASP Ind. & Com. LTDA, Curitiba, Brazil; ^4^Department of Technology, Faculdade de Ciências Agrárias e Veterinárias, UNESP – Universidade Estadual Paulista, Jaboticabal, Brazil; ^5^INCT/CA – UFV, Department of Animal Science, Viçosa, Brazil; ^6^Embrapa Southeast Livestock, São Carlos, Brazil

**Keywords:** archaea diversity, beef cattle, enteric methane emission, nitrate, rumen bacteria diversity, volatile fatty acids

## Abstract

This study investigated the long-term effects (13 months) of encapsulated nitrate supplementation (ENS) on enteric methane emissions, rumen fermentation parameters, ruminal bacteria, and diversity of archaea in grazing beef cattle. We used a total of thirty-two Nellore steers (initial BW of 197 ± 15.3 kg), 12 of which were fitted with rumen cannulas. For 13 months, the animals were maintained in 12 paddocks and fed a concentrate of ground corn, soybean meals, mineral supplements, and urea (URS) or encapsulated nitrate (EN) containing 70 g of EN/100 kg of BW (corresponding to 47 g NO_3_^-^/100 kg BW). Encapsulated nitrate supplementation resulted in similar forage, supplement and total DMI values as URS (*P* > 0.05), but ENS tended to increase (+48 g/d; *P* = 0.055) average daily weight gain. Daily reductions in methane emissions (-9.54 g or 18.5%) were observed with ENS when expressed as g of CH_4_/kg of forage dry matter intake (fDMI) (*P* = 0.037). Lower concentrations of NH_3_-N and a higher ruminal pH were observed in ENS groups 6 h after supplementation (*P* < 0.05). Total VFA rumen concentration 6 h (*P* = 0.009) and 12 h after supplementation with EN resulted in lower acetate concentrations in the rumen (*P* = 0.041). Steers supplemented with EN had a greater ruminal abundance of *Bacteroides, Barnesiella, Lactobacillus*, *Selenomonas, Veillonella, Succinimonas, Succinivibrio*, and *Duganella* sp. (*P* < 0.05), but a lower abundance of *Methanobrevibacter* sp. (*P* = 0.007). Strong negative correlations were found between daily methane emissions and Proteobacteria, Erysipelotrichaceae, Prevotellaceae, and *Roseburia*, *Kandleria*, *Selenomonas*, *Veillonella*, and *Succinivibrio* sp. (*P* < 0.05) in the rumen of ENS steers. Encapsulated nitrate is a feed additive that persistently affects enteric methane emission in grazing steers, thereby decreasing *Methanobrevibacter* abundance in the rumen. In addition, ENS can promote fumarate-reducer and lactate-producer bacteria, thereby reducing acetate production during rumen fermentation.

## Introduction

Global methane emissions from ruminant livestock has increased by 332% since the 1980s, prompting serious concerns about the increasing environmental cost of livestock production (Dangal et al., 2017). Enteric methane production also represents an energy loss of nearly 12% of the gross energy of feed (Johnson and Johnson, 1995). Therefore, the mitigation of methane emissions has become a research priority in ruminant nutrition.

Nitrates (NO_3_^-^) are effective methane inhibitors and a potential non-protein nitrogen source for cattle, acting as an H_2_ sink and adding ammonia-based nitrogen to the rumen (Nolan et al., 2010; Zhao et al., 2015). When microbial NO_3_^-^ reduction is incomplete, rumen nitrite accumulation could result in host poisoning (Leng, 2008). Nitrate encapsulation enables the slow rumen release of NO_3_^-^ in the rumen reducing the risk of toxicity (Mamvura et al., 2014). It has been used as a substitute for supplementary urea or true protein sources in beef cattle, dairy cows, sheep, and lambs, resulting in a reduction in enteric methane production and improvement in the utilization of feed energy in some cases, with variable effects on rumen fermentation and nutrient digestibility (Van Zijderveld et al., 2010, 2011; El-Zaiat et al., 2014; Lee et al., 2015a; Olijhoek et al., 2016).

Rumen microorganisms play an important role in host energetic and protein metabolism, and rumen compound detoxification (Weimer, 1998). Methane is produced exclusively by methanogenic archaea, but all rumen microorganisms participate in methanogenesis in a direct or indirect way (Yang et al., 2016) following the excessive production of H_2_, which needs to be removed as CH_4_ (Martin et al., 2010).

Some feed additives (i.e., monensin and bromochloromethane) reduce enteric methane emissions in the short-term but lose effectiveness in the long-term because of microbial adaptations to the compounds (Lee and Beauchemin, 2014). Short-term experiments have shown that the addition of nitrate into ruminant diets could affect archaea, protozoa, and fungi abundance (Asanuma et al., 2015), stimulate nitrate- and nitrite-reducing bacteria, such as *Campylobacter fetus*, *Mannheimia succiniciproducens*, and *Selenomonas ruminantium* (Lin et al., 2013; Asanuma et al., 2015; Zhao et al., 2015), and promote growth of ruminal cellulolytic bacterial species (Lin et al., 2013; Zhao et al., 2015). However, there remains a lack of knowledge on the responses of rumen bacteria and archaea diversity to the long-term use of encapsulated nitrate (EN) in grazing cattle.

We hypothesized that EN induces change the composition of bacterial and archaeal communities, resulting in lower methane emissions in grazing Nellore steers after only 13 months of use. Thus, the objective of this study was to investigate the effects of the long-term use of EN supplementation (ENS) on enteric methane emissions, rumen fermentation parameters, and ruminal bacteria and archaea diversity in grazing beef cattle.

## Materials and Methods

This study was carried out in accordance with the recommendations of the Brazilian College of Animal Experimentation (COBEA – Colégio Brasileiro de Experimentação Animal) guidelines. The protocol was approved by the Ethics, Bioethics, and Animal Welfare Committee (CEBEA – Comissão de Ética e Bem Estar Animal) of the School of Agricultural and Veterinary Studies (FCAV) of the Universidade Estadual Paulista (UNESP), Jaboticabal campus, Brazil (Protocol number 11242/14).

### Animals and Grazing Area

The experiment was conducted at the São Paulo Agribusiness Technology Agency (APTA), Alta Mogiana regional pole, at Colina, Brazil (20° 43′ 05″ S latitude and 48° 32′ 38″ W longitude), from July/2014 to September, 2015. The experiment was part of a larger study conducted over three time periods: 135 days of the dry season (July to November 2014), 168 days of the rainy season (November 2014 to May 2015) and 104 days of the finishing phase (May to September 2015), however, only data from the finishing phase are presented in this study.

We used 32 Nellore steers of (8 ± 1 months old and 197 ± 15.3 kg; final BW 483 ± 33 kg) of which 12 were fitted with silicone-type ruminal cannulas (10 cm of i.d.; Kehl^®^, São Carlos, Brazil). The cannulas were used to record the effects of EN on supplement ruminal fermentation parameters and rumen microbiology. The 20 non-cannulated animals were using to estimate standard methane emissions during the finishing phase. Ruminal cannulation was conducted 1 month prior to the onset of the experiment under xylazine sedation and local anesthesia with lidocaine hydrochloride; all efforts were made to minimize suffering.

Animals were randomly distributed in 12 paddocks, with 1 cannulated steer in each paddock. The pastures used during the dry season contained *Panicum maximum* “Tanzânia” divided in 12 paddocks, 3.0 ha each. During the rainy season and finishing phase, animals were kept in a pasture of *Brachiaria brizantha* “Marandu,” divided by electric fencing divided into 12 paddocks with an area of 2.2 ha each. Each paddock was served by a 1000 L capacity automatic metal water trough and collectives covered plastic feeders to provide the supplement.

The grazing method adopted was that of continuous stocking with a variable stocking rate using the put and take technique (Mott and Lucas, 1952). Estimates of forage mass (Supplementary Table S1) were performed using the double sampling method (Sollenberger and Cherney, 1995) and its nutritional value was estimated using the simulated grazing method (De Vries, 1995). Samples were hand-plucked every 28 day simultaneously with herbage mass sampling (20 average spots heights each paddock), dried at 55 ± 5°C to a constant weight under forced air and then ground through a 1 mm screen in a shear mill (Thomas-Wiley Laboratory Mill Model 4, H. Thomas Co.) for chemical analyses.

### Experimental Supplements

Animals were supplemented with concentrate composed of ground corn, soybean meal, mineral supplement, and urea [Urea supplement (URS)] or EN (Nitrate supplement) containing 70 g of EN/100 kg of BW, corresponding to 47 g NO_3_^-^/100 kg BW. Thus, 0.7, 0.5, and 1.5% EN of BW was made available during the dry and rainy seasons, and finishing phase, respectively (Supplementary Table S1). In the urea supplements, limestone was used to maintain supplements with similar Ca concentrations and urea inclusion was used to represent the protein equivalent to EN.

The source of nitrate was the double salt of calcium ammonium nitrate decahydrate [5Ca(NO_3_)_2_⋅NH_4_NO_3_⋅10H_2_O]. Nitrate doses were maintained until the slaughter of the animals, selected based on methane mitigation capacity, which was approximately 12.42 g of CH_4_/100 kg BW (Van Zijderveld et al., 2010). The EN was manufactured by GRASP Ind. & Com. LTDA (Curitiba, Brazil) and EW Nutrition GmbH (Visbek, Germany) and contained 85.6% of dry matter (DM; as fed basis), 16% N, 19.6% Ca, and 67.1% NO3.

### Intake Estimation

Forage and supplement intake and fecal excretion were estimated for cannulated animals during the finishing phase 398 to 406 days and using three markers. Chromium oxide (Cr_2_O_3_), titanium dioxide (TiO_2_), and indigestible NDF (iNDF) were used to estimate the excretion of fecal matter (as dry weight), supplement and forage intake, respectively.

To estimate fecal excretion, 10 g per animal/day of the external indicator (Cr_2_O_3_) was placed directly in the rumen for 9 days (5 days before sampling and 4 days of fecal excretion sampling periods). Fecal samples were collected directly from the rectum, once daily alternating at the following times: 0700, 1000, 1300, and 1600 h.

Fecal samples were weighed and dried in a forced-air-circulation oven at 55°C for 72 h, and ground in a Wiley mill (Thomas Scientific, Swedesboro, NJ, United States) to pass through a 1 mm screen. For each animal, in each sampling period, a fecal-composite sample based on the pre-dried weight and chromium oxide was measured in an atomic absorption spectrophotometer (Czarnocki et al., 1961).

Fecal excretion was calculated according to the following equation: Fecal excretion = [chromium oxide supplied (g/day)]/[fecal chromium oxide concentration (g/g MS)] (Smith and Reid, 1955).

To estimate dry matter intake (DMI) of the supplement, TiO_2_ was added as an external marker to the supplement at a rate of 10 g/day of TiO_2_ per animal (10 g/day × no. of animal/paddock) for 9 days, 6 days to stabilize the fecal excretion marker, and 3 days for sample collection (Titgemeyer et al., 2001). Fecal samples were taken simultaneously with fecal excretion procedures. Feces were dried at 55 ± 5°C for 72 h, to a constant weight, pooled based on animal species, ground, and digested using sulfuric acid. A standard curve was prepared by adding 0, 2, 4, 6, 8, and 10 mg of TiO_2_ to the samples, and it was read using a spectrophotometer at 410 nm as described by Myers et al. (2004). Individual supplement intake was estimated using the following equation: Supplement DMI = [g of TiO_2_/g of feces × fecal excretion g/d]/g TiO2/g of supplement.

The forage DMI was estimated using an internal marker iNFD, determined after ruminal incubation for 288 h (Valente et al., 2011). Forage DMI was estimated from the fecal output of the internal marker corrected for the supplement contribution as follows:

Forage DMI = [fecal excretion g/d × (*i*MF) - DMI of supplement × (*i*MS)]/[*i*MH], where *i*MF, *i*MS, and *i*MH are the concentrations of the internal marker in feces, supplement, and forage, respectively. Total DMI was obtained by the addition of forage and supplement DMI.

Samples were analyzed for DM (Method 934.01) and ash (Method 942.05), according to AOAC (1990).

### Methane Emissions and Body Weight Gain

Methane emissions were estimated from 20 non-cannulated animals (10 per experimental supplement) during the finishing phase (day 391 to 396). Animal methane emission measurements were performed using the tracer gas sulfur hexafluoride (SF6) technique (Johnson et al., 1994). Briefly, CH_4_ flow released by the animal was calculated in relation to the flow of SF_6_, measured from the SF_6_ release rate from a permeation capsule lodged in the rumen and from the concentrations of CH_4_ and SF_6_ in gas samples (Johnson et al., 2007).

Two weeks before methane collected, capsules containing SF_6_, with a known permeation rate (899.82 and 872.60 ng/min for urea and EN treatments, respectively) were placed in the rumen via the esophagus, and they remained in the rumen throughout the experimental period. Three weeks prior to collection, animals were acclimated to the PVC collection containers. Expired gasses were collected continuously (24 h periods) into evacuated PVC containers during the 5 days of collection. Daily background air samples were collected at two points of experimental area using the same procedures above.

Expired gasses and background samples were analyzed for concentrations of CH_4_ (ppm, parts per million by volume) and SF_6_ (ppt, parts per trillion by volume) using gas chromatography equipped with a flame ionization detector and electron capture. Daily CH_4_ emissions were calculated from the specific SF_6_ permeation rates and the CH_4_/SF_6_ ratio of concentrations in breath samples, after adjustment for background gas concentrations (Johnson et al., 1994).

To determine average daily gain, animals were weighed at the beginning and end of the trial, after 16 h of fasting from solids and liquids. Intermediate weighing (every 28 days, no fasting) was also performed to adjust supplement delivery and the dosage of EN.

### Rumen Fermentation Parameters

Rumen fermentation parameters were measured in cannulated animals over 2 days during the finishing phase (345 and 407 days) at 0, 6, 12, and 18 h after supplementation. Approximately 100 mL of ruminal fluid was recovered after filtration and placed through double layer cheesecloth. Then the pH was measured using a digital pH meter (DM-1069 22, Digimed, São Paulo, Brazil). Two 50 mL aliquots were stored at -20°C and later used to determine ammonia nitrogen (NH_3_-N) and volatile fatty acid (VFA) concentrations. The NH_3_-N aliquot was acidified with 1 mL of H_2_SO_4_ for analysis using the colorimetric method (Weatherburn, 1967). The VFA concentration was quantified by gas chromatography (GC Shimadzu model 20–10, with automatic injection; Shimadzu Corporation, Kyoto, Japan) using a SP-2560 capillary column (30 m × 0.25 mm diameter, 0.02 mm thick; Supelco, Bellefonte, PA, United States) according to the method described by Erwin et al. (1961).

### Ruminal Microorganism

Rumen microorganism in cannulated animals were studied during the finishing phase. Samples of ruminal content were collected on day 407, early in the morning, before supplementation. Samples of approximately 100 g per animal (a mix of liquid and solid) from the dorsal, central, and ventral regions of the rumen were collected through the ruminal cannula, immediately placed into a thermo-box cooled to 4°C, and transferred to the laboratory for DNA extraction.

The samples were weighed and immediately processed to obtain a bacterial pellet as described by Granja-Salcedo et al. (2017). A Fast DNA SPIN Kit for Soil (MP Bio^®^, Biomedicals, Illkirch, France) extraction kit was used to extract metagenomic DNA from 250 mg of bacterial pellet according to the manufacturer’s instructions. The DNA concentrations were measured fluorometrically (Qubit^®^ 3.0, kit Qubit^®^ dsDNA Broad Range Assay Kit, Life Technologies, Carlsbad, CA, United States) and DNA purity was assessed spectrophotometrically (NanoDrop^®^ ND-1000 Spectrophotometer, Thermo Fisher Scientific, Waltham, MA, United States) at A260/A230 and A260/A280 nm. DNA integrity was determined by agarose gel electrophoresis using a 0.8% (w/v) gel, and subsequent staining with SYBR Safe DNA Gel Stains (Invitrogen, Carlsbad, CA, United States).

A PCR was employed to amplify the V3 and V4 regions of the 16S ribosomal RNA gene 16S rRNA for bacteria (Caporaso et al., 2011). Each sample was amplified in duplicates, and each PCR reaction mixture (20 μL final volume) contained 20 ng of metagenomic DNA, 10 μM of each forward and reverse primers, 1.25 mM of magnesium chloride, 200 μM of dNTP mix (Invitrogen, Carlsbad, CA, United States), 1.0 U platinum Taq DNA polymerase high fidelity (Invitrogen, Carlsbad, CA, United States), high fidelity PCR buffer [1X], and milli-Q water. Reactions were held at 95°C for 3 min to denature the DNA, with amplification proceeding for 30 cycles at 95°C for 30 s, 53.8°C for 30 s, and 72°C for 45 s; a final extension of 10 min at 72°C was added to ensure complete amplification.

The expected fragment length of PCR products was verified by agarose gel (1%) electrophoresis, and the amplicon size was estimated by comparison with a 1 kb plus DNA ladder (1 kb plus DNA ladder, Invitrogen, Carlsbad, CA, United States). The PCR fragments were purified using the Zymoclean^TM^ Gel DNA Recovery kit following the manufacturer’s instructions. Composite samples for sequencing were created by combining equimolar ratios of amplicons from the duplicate samples. Sequencing was performed using the Ion Torrent Personal Genome Machine (Life Technologies, Carlsbad, CA, United States) using the Ion 314^TM^ Chip Kit v2.

Sequence data were processed, removing adapters using Scythe 0.991^1^ and Cutadapt 1.7.1 (Martin, 2011). Sequence trimming was carried out by selecting sequences over 200 bp in length with an average quality score greater than 20 based on Phred quality, and duplicate reads were removed using the Prinseq program (Schmieder and Edwards, 2011). We used the Quantitative Insights into Microbial Ecology (QIIME) software package version 1.9.1 to filter reads and determine Operational Taxonomic Units (OTUs) as described in Caporaso et al. (2010). The Usearch algorithm was used to cluster the reads OTUs with a 97% cutoff, and to assign taxonomy using the Ribosomal Database Project (RDPII) version 10 (Cole et al., 2014). Bacterial sequences were de-noised and suspected chimeras were removed using the OTU pipe function within QIIME. Sequence data were summarized at the phylum, class, and family levels; In addition, Alpha_diversity.py in QIIME was used to calculate ACE, Chao1, Shannon, and Simpson indices. Principal coordinate analyses (PCoA) were conducted to evaluate differences in community structure among experimental groups (β-diversity). PCoA was generated with unweighted Unifrac distance (Lozupone et al., 2006) using the R package vegan version 2.0–10.

### Statistical Analyzes

Statistical analyses were performed using R Software version 3.4.3 (R Core Team, 2015). Initially, mathematical assumptions of data were tested (Shapiro–Wilk and Bartlett tests).

Data of intake and methane emissions were compared between treatments using an ANOVA as a completely randomized design, with 2 treatments (URS and ENS) and 6 (intake) or 10 (methane and weight gain) repeats. The model included treatments as fixed effects, and the residues corresponding to the model as random effects.

Rumen pH, NH_3_-N, and VFA were compared between treatments and time by a repeated measures ANOVA using a completely randomized design. The model included fixed effects of treatments, sampling time and its interaction, and the random effects of residues of treatments and residues corresponding to the model.

Bacterial and Archaea data were compared between experimental groups using the Wilcoxon test. Spearman’s rank correlations were used to investigate the relationship between bacterial and archaeal phylum or genera and DMI, CH_4_ emissions, and rumen fermentation parameters, and a correlation plot was performed only with significant correlations using the corrplot library in R. Statistical significance was set to *p* < 0.05 and a tendency of difference was declared at *p* < 0.10.

## Results

### Intake, Methane Emissions, and Weight Gain

Encapsulated nitrate supplementation (ENS) resulted in similar forage, supplement, and total DMI values than URS, when expressed in terms of kg per day and body weight (Table 1; *P* > 0.05). Daily methane emission was not affected when expressed as g of CH_4_ per kg of total dry matter intake (tDMI) or as g of CH_4_ per kg of supplement dry matter intake (sDMI) (*P* > 0.05). However, when expressed as g of CH_4_ per day, we found lower methane emissions (-28.62 g or 10.55%) in animals supplemented with EN (*P* = 0.085). We found a similar reduction (-9.54 g or 18.5%) when daily methane emissions were expressed as g of CH_4_ per kg forage dry matter intake (fDMI) (*P* = 0.037). In addition, ENS increased (+43 g/d) the average daily weight gain (*P* = 0.055), resulting in a higher final BW (+30.7 kg) when compared to URS (*P* = 0.032).

**Table 1:** Intake, methane emissions and weight gain in grazing Nellore Steers a after 13 months of supplementation with encapsulated nitrate (ENS) or Urea (URS).

	Treatment			*P*-value
	URS	ENS	SEM	
***Intake, kg/d***				
Total	13.51	13.19	0.693	0.833
Forage	5.27	5.79	0.550	0.674
Supplement	8.24	7.40	0.375	0.302
***Intake, % of BW***				
Total	2.78	2.75	0.117	0.910
Forage	1.04	1.22	0.097	0.387
Supplement	1.73	1.55	0.081	0.341
CH_4_, g/d	271.25	242.63	14.879	0.085
CH_4_, g/kg tDMI	20.07	18.40	1.057	0.498
CH_4_, g/kg fDMI	51.47	41.93	2.273	0.037
CH_4_, g/kg sDMI	32.90	32.79	1.097	0.821
CH_4_, g/kg BWG	384.43	234.37	11.374	0.025
ADG, kg	0.705	0.748	0.014	0.055
Final BW	490.4	521.1	18.031	0.032

*SEM, standard error of mean; tDMI, total dry matter intake, fDMI, forage dry matter intake, sDMI, supplement dry matter intake, BWG, body weight gain, ADG, average daily weight gain.*

### Rumen Fermentation Parameters

**FIGURE 1:**
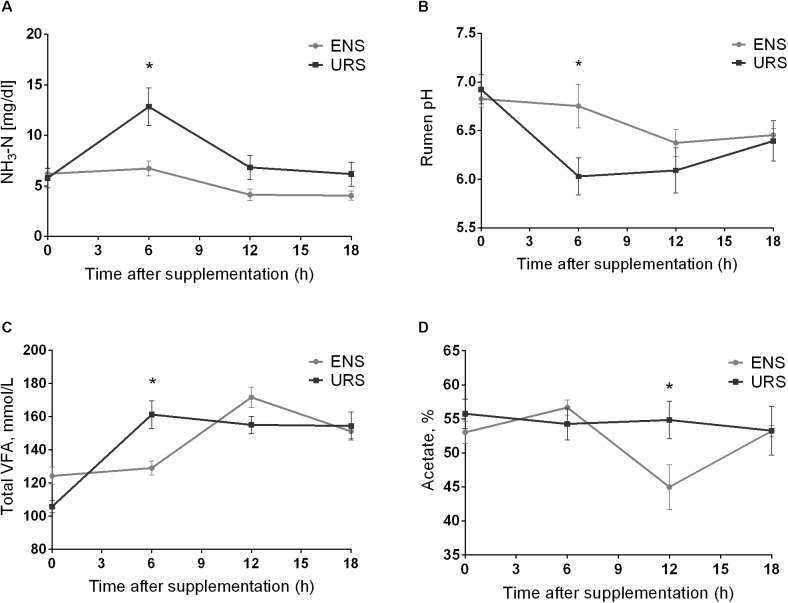
Mean and standard error of mean of ruminal ammonia (NH_3_-N) concentration **(A)**, pH values **(B)**, total volatile fatty acid (VFA) rumen concentration **(C)** and Acetate proportions **(D)** during a 18 h period in grazing Nellore Steers after a long time (13 months) of supplementation with encapsulated nitrate (ENS) or Urea (URS). ^∗^supplement effect and time effect interaction (*P* < 0.05) as obtained with Tukey’s test.

Rumen propionate, *iso*-butyrate, butyrate, and valerate proportions were not affected by the supplements tested (Table 2; *P* > 0.05). However, there was an interaction effect between supplements and time at different NH_3_-N ruminal concentrations, rumen pHs, total VFA rumen concentrations, and acetate proportions (Table 2; *P* < 0.05). Steers fed ENS had the lowest NH_3_-N ruminal concentrations, 6 h after supplementation (Figure 1A; *P* = 0.010). Rumen pH values were higher 6 h after ENS (Figure 1B; *P* = 0.033), whereas total VFA rumen concentrations (Figure 1C; *P* = 0.009) and acetate concentrations decreased 6 and 12 h after supplementation, respectively (Figure 1D; *P* = 0.041). Ruminal proportions of *iso*-valeric acids were higher after URS (Table 2; *P* < 0.001).

**Table 2:** Rumen fermentation parameters in grazing Nellore Steers after 13 months of supplementation with encapsulated nitrate (ENS) or Urea (URS).

	Treatment		SEM	*P*-value		
	URS	ENS		S	T	S x T
pH	6.359	6.601	0.077	0.028	< 0.001	0.053
NH_3_-N, mg/dL	8.236	5.272	0.517	0.001	< 0.001	0.045
Total VFA, mmol/L	144.378	143.611	5.949	0.842	< 0.001	<0.001
Acetate, %	54.766	51.862	1.677	0.015	0.122	0.041
Propionate, %	20.462	21.502	0.994	0.313	0.060	0.182
*Iso*-butyrate, %	8.464	8.568	0.386	0.721	< 0.001	0.107
Butyrate, %	10.434	10.856	0.561	0.922	0.003	0.358
*Iso*-valerate, %	2.075	1.422	0.128	< 0.001	0.005	0.273
Valerate, %	3.271	3.449	0.221	0.547	0.007	0.594
A:P	2.676	2.412	0.711	0.169	0.599	0.525

*VFA, volatile fatty acid; A: P, acetate propionate; SEM, standard error of mean; S, supplements effect; T, time effect.*

### Ruminal Microorganism

For all amplicons, good’s coverage of all samples was >0.996. The number of generated sequences after filtering analyses, observed OTUs, richness (Chao1 and ACE), and diversity estimators (Shannon Wiener and Simpson) by rumen bacteria and archaea populations were similar between ENS and URS groups (Supplementary Table S2; *P* > 0.05). Comparisons of bacterial communities by principal coordinate analysis (PCoA) using the weighted Unifrac distance (Supplementary Figure S1), explained 56.14% of the variation in the data and showed a tendency separation between ENS and URS (*P* = 0.061).

**FIGURE 2:**
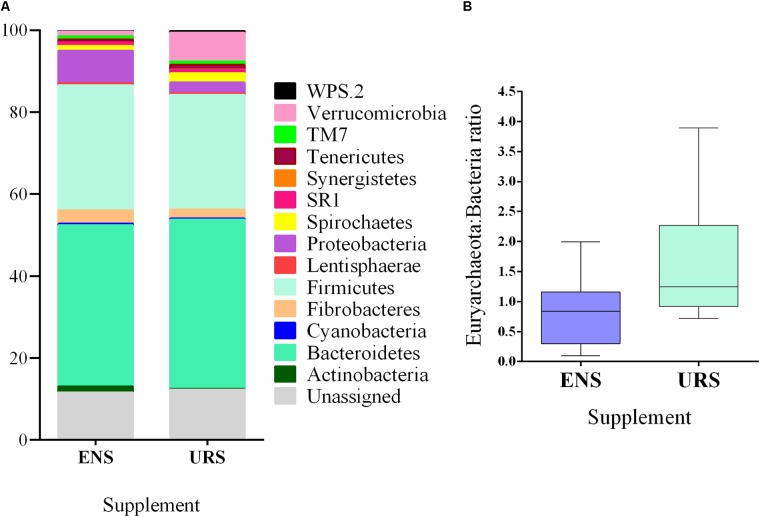
Bacterial abundance at phylum level **(A)** and Euryarchaeota: Bacteria ratio **(B)** in grazing Nellore Steers after a long time (13 months) of supplementation with encapsulated nitrate (ENS) or Urea (URS).

Fourteen phyla were identified (Figure 2A and Supplementary Table S3) and 14.18 and 17.30% of the sequences could not be classified at the phylum level to ENS and URS, respectively. Firmicutes and Bacteroidetes were the most abundant phyla and accounted for >64% of the total bacterial community in all samples sequenced. In the Archaea community, Euryarchaeota was the only phyla identified.

The abundance of Actinobacteria, Fibrobacteres, Firmicutes, and Proteobacteria was higher in the rumen of ENS than URS supplemented steers (Supplementary Table S3; *P* < 0.05). In contrast, Verrucomicrobia phylum abundance (Figure 2A; *P* = 0.0031) and Euryarchaeota: Bacteria ratios were lower in ENS steers (Figure 2B; *P* = 0.0285).

Steers supplemented with ENS had a greater ruminal abundance of *Betaproteobacteria* and *Epsilonproteobacteria* (*P* < 0.05). Increases in the abundance of *Bacteroides, Barnesiella, Lactobacillus*, *Selenomonas, Veillonella, Succinimonas, Succinivibrio*, and *Duganella* sp. were also observed in these animals (Table 3; *P* < 0.05). In contrast, *Paraprevotella* sp. (*P* = 0.0214) had a greater ruminal abundance in URS supplemented steers.

**Table 3:** Median and interquartile range of the rumen methanogens and bacterial abundance at genera in grazing Nellore Steers after 13 months of supplementation with encapsulated nitrate (ENS) or Urea (URS).

			Treatment		*P*-value
Domain	Phylum	Genera	URS	ENS	
Bacteria	Actinobacteria	*Olsenella*	0.045 ± 0.02	0.060 ± 0.13	0.286
	Cyanobacteria	*GpXII_Other*	0.238 ± 0.40	0.342 ± 0.22	0.555
	Bacteroidetes	*Bacteroides*	0.018 ± 0.01	0.339 ± 0.27	0.016
		*Marinilabiliaceae_Other*	0.019 ± 0.08	0.100 ± 0.05	0.176
		*Barnesiella*	4.499 ± 2.88	10.113 ± 5.77	0.038
		*Paraprevotella*	0.104 ± 0.01	0.153 ± 0.02	0.413
		*Prevotella*	28.263 ± 9.94	19.905 ± 3.50	0.021
		*Prevotellaceae_Other*	0.053 ± 0.04	0.112 ± 0.10	0.555
	Fibrobacteres	*Fibrobacter*	2.181 ± 1.06	3.283 ± 2.22	0.061
	Firmicutes	*Lactobacillus*	0.009 ± 0.01	0.645 ± 0.31	0.019
		*Mogibacterium*	0.213 ± 0.20	0.218 ± 0.16	0.905
		*Eubacterium*	0.126 ± 0.03	0.234 ± 0.08	0.436
		*Blautia*	0.288 ± 0.04	0.283 ± 0.05	0.914
		*Clostridium_XlVa*	0.019 ± 0.01	0.029 ± 0.01	0.176
		*Lachnospiraceae_Other*	0.655 ± 0.01	0.448 ± 0.42	0.412
		*Pseudobutyrivibrio*	0.101 ± 0.34	0.032 ± 0.06	0.384
		*Roseburia*	0.094 ± 0.05	0.146 ± 0.71	0.728
		*Clostridium_IV*	0.197 ± 0.08	0.117 ± 0.02	0.111
		*Ruminococcus*	0.589 ± 0.22	0.631 ± 0.31	0.803
		*Kandleria*	0.094 ± 0.03	0.045 ± 0.02	0.730
		*Mitsuokella*	0.733 ± 0.24	0.756 ± 4.28	0.063
		*Selenomonas*	1.742 ± 0.24	4.283 ± 0.75	0.009
		*Veillonella*	0.075 ± 0.05	0.361 ± 0.91	0.014
	Proteobacteria	*Betaproteobacteria^§^*	0.014 ± 0.00	0.196 ± 0.05	0.037
		*Gammaproteobacteria^§^*	0.429 ± 0.04	0.531 ± 0.07	0.453
		*Epsilonproteobacteria^§^*	0.011 ± 0.01	0.093 ± 0.01	0.023
		*Geobacteraceae_Other*	0.037 ± 0.04	0.084 ± 0.18	0.555
		*Succinimonas*	0.401 ± 0.15	2.369 ± 0.24	0.041
		*Succinivibrio*	2.281 ± 0.57	4.685 ± 0.49	0.019
		*Duganella*	0.001 ± 0.00	0.149 ± 0.30	0.062
	Tenericutes	*Anaeroplasma*	0.094 ± 0.14	0.063 ± 0.02	0.285
	Spirochaetes	*Sphaerochaeta*	0.106 ± 0.15	0.159 ± 0.11	0.905
		*Treponema*	2.098 ± 1.74	1.032 ± 0.93	0.730
Archaea	Euryarchaeota	*Methanobacterium*	0.013 ± 0.01	0.019 ± 0.02	0.156
		*Methanobrevibacter*	1.205 ± 0.95	0.579 ± 0.81	0.007
		*Methanomassiliicoccus*	0.021 ± 0.01	0.035 ± 0.02	0.713

*^§^ Class level. Differences were considered significant at *p* < 0.05 using the Wilcoxon test.*

In the Archaea community, the abundance of *Methanobrevibacter* sp. (Table 3; *P* = 0.007) was lower in the rumen of ENS than URS supplemented steers.

**FIGURE 3:**
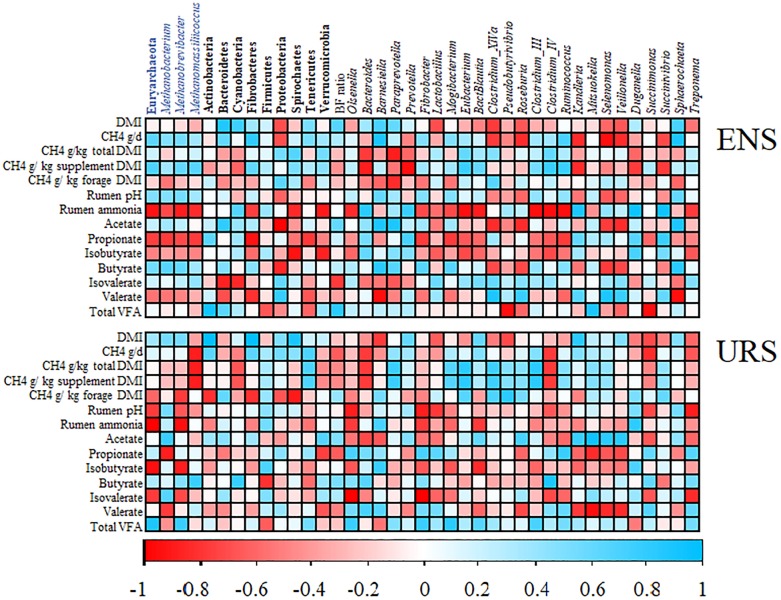
Correlation analysis between dry matter intake, methane emissions, rumen fermentation parameters and relative abundances of ruminal microbial taxa in encapsulate nitrate supplementation (ENS) and urea supplementation (URS) groups. Spearman’s correlation values for phylum/genera across all samples was performed and only significant correlations (*P* < 0.10) for at least one of the analyzed variables are shown. Names in blue and black indicate archaeal and bacteria taxa, respectively. Names in bold and italics indicate phylum and genera level, respectively. CH_4_, methane emissions; DMI, dry matter intake expresses as kg per day; VFA, volatile fatty acids.

A Positive correlation between CH_4_ emissions (g/day and g/kg of supplement DMI) and Euryarchaeota, and *Methanobacterium, Methanobrevibacter*, and *Methanomassiliicoccus* sp. was observed in the ENS group (Figure 3 and Supplementary Table S4). However, the strongest negative correlations were found between daily CH4 emissions (g/d) and bacteria for sequences belonging to the Proteobacteria, and *Clostridium_XlVa*, *Roseburia*, *Kandleria*, *Selenomonas*, *Veillonella*, and *Succinivibrio* sp. (*r* ≥-0.70, *P* ≤ 0.05) in the rumen of ENS supplemented steers (Figure 3 and Supplementary Table S4). CH_4_ emissions (g/kg of supplement DMI), also negatively correlated with *Bacteroides*, *Prevotella*, *Kandleria*, *Duganella*, and *Succinivibrio* sp. (*r* ≥-0.77, *P* ≤ 0.04); however, the same microorganism were positively correlated with ammonia concentrations in the ENS group (*r* ≥ 0.83, *P* ≤ 0.03).

For the URS group, *Methanomassiliicoccus*, *Bacteroides*, and *Clostridium_lV* sp. were negatively correlated (*r* ≥-0.77, *P* ≤ 0.04) with daily CH4 emissions in g/day, g/kg of total DMI, and g/kg of supplement DMI (Figure 3 and Supplementary Table S4). In addition, *Succinimonas* were negatively correlated (*r* = -0.83, *P* = 0.03) with daily CH4 emissions (g/day).

Euryarchaeota and *Methanobacterium, Methanobrevibacter*, and *Methanomassiliicoccus* sp. negatively correlated (*r* > -0.71, *P* < 0.05) with rumen ammonia and propionate proportions in the ENS group (Figure 3 and Supplementary Table S4). Similarly, negative correlation were observed between rumen ammonia and Fibrobacteres, Spirochaetes, Verrucomicrobia, *Bautia*, *Clostridium_III*, *Clostridium_IV*, *Eubacterium*, *Fibrobacter*, *Mogibacterium*, *Olsenella*, *Ruminococcus*, and *Treponema* sp. (*r* ≥-0.71, *P* ≤ 0.05).

## Discussion

The low methane emissions associated with a lower Euryarchaeota: Bacteria ratio and abundance of *Methanobrevibacter* sp. in the rumen of ENS steers after 13 months, supported our prediction that EN is a feed additive that affects enteric methane emissions persistently. In addition, increases in bacterial abundance with reported fumarate reduction capacity and lactate production in the rumen of ENS steers highlights changes in rumen fermentation pathways stimulated by nitrate supplementation. Thus, the hypothesis that ENS induces changes in bacterial and archaeal community compositions, thereby modulating lower methane emissions in grazing Nellore steers long-term use was accepted.

After 13 months, EN intake was 358.16 g/d, resulting in a 2.7% EN concentration in the diet. Even when possible changes in diet organoleptic properties by NO_3_^-^ could decrease feed intake in cattle (Lichtenwalner et al., 1973; Lee et al., 2015b), we observed similar forage, supplement, and total DMI pattern between ENS and URS groups. These results are similar to that of previous studies on cattle fed high forage diets and supplemented with 2.5 and 3.0% of EN (Lee et al., 2015a, 2017a). Similarly, Lee et al. (2017b) believed that it could be potentially beneficial to encapsulate NO_3_^-^ to improve its organoleptic properties when incorporated it into a backgrounding diet in cattle.

The methane reduction of -28.62 g of CH_4_/d (-10.55% of URS group) and -9.54 g of CH_4_/kg fDMI (-18.5% of URS group) by ENS, verified the effects of the H_2_ sink of nitrate in the rumen. However, nitrate mitigation was lower than expected; stoichiometrically, 100 g of NO^3-^ should lower methane emissions by 25.8 (Van Zijderveld et al., 2010). In our study, the intake of 358.16 g of EN that supplied 240.47 g of NO_3_^-^ should have reduced 62.04 g of CH_4_/d, but we only observed an efficiency of 46.14%. This result is in line with that of Newbold et al. (2014) who found 49% efficiency in beef cattle, but lower efficiencies were observed by Van Zijderveld et al. (2011); Hulshof et al. (2012), and Olijhoek et al. (2016). In most cases, inefficient methane mitigation is observed because of the incomplete reduction of nitrate to nitrite or nitrite to ammonia (Newbold et al., 2014) or the redirection of electrons to propionate production rather than methanogenesis (Van Zijderveld et al., 2011).

Animal performance was not affected by ENS, as shown by Lee et al. (2017b), which suggests a reduction in toxicity when NO_3_^-^ is encapsulated. In addition, we found that ENS increased ADG (+43 g/d) resulting in +30.7 kg of final BW. Nitrate may improve animal performance, once the complete reduction of NO_3_^-^ to NH_3_-N yields more energy than the conversion of CO_2_ to CH_4_ (Ungerfeld and Kohn, 2006). In feedlot trials, such slight improvements were observed by Pereira et al. (2013) and Lee et al. (2017b) using up 3.0 and 1.25% of EN in dietary DM. However, when the basal diet had a high-forage content ENS had no effect on ADG (El-Zaiat et al., 2014; Lee et al., 2017a). These findings suggest that the effects of ENS on animal performance is dependent on several factors such as basal diet.

Our analysis of the ruminal microbiota indicates that ENS modulated the profiles of rumen archaea communities to lower methane production over time, suggesting that there was no microbial adaptation to nitrate compounds. We observed a lower Euryarchaeota: bacteria ratio and lower abundance of *Methanobrevibacter* sp. in the rumen of ENS steers. Euryarchaeota and *Methanobrevibacter* sp. were positively correlated with methane emissions. Similarly, Wallace et al. (2014, 2015) found an association between the abundance of total archaea and *Methanobrevibacter* sp. and higher methane emissions in cattle. It is possible that nitrate or its reduced forms might be toxic to methanogens (Iwamoto et al., 2002). *In vitro* trials have shown that the nitrate ester is highly specific toward archaea growth inhibition and that *Methanobrevibacter* *ruminantium* growth inhibition required 100 times lower concentrations (Duin et al., 2016). In addition, rumen H_2_ deficiency (energy source for methanogens) might reduce the archaea population in the rumen (Lee and Beauchemin, 2014), resulting in the negative correlation observed between archaea populations and rumen ammonia concentrations in ENS groups. This suggests that the use of H_2_ in the reduction of nitrate to ammonia in the rumen, was one of the mechanisms used to reduce ruminal methanogen populations.

Different archaea species could benefit from changes in H_2_ concentrations or could respond differently to CH_4_ substrate availability (Kittelmann et al., 2014). We found that ENS decreased *Methanobrevibacter* abundance, whereas *Methanobacterium* and *Methanomassiliicoccus* populations remained unaffected. *Methanomassiliicoccus* sp. belong to the Thermoplasmata class, a very poorly characterized archaea group. *Methanobrevibacter* and *Methanobacterium* belong to the same family, however, *Methanobrevibacter* is the most abundant methanogen found in the rumen (Janssen and Kirs, 2008); this genus does not contain homologues of the *mta* genes required for methanol utilization in other methanogens (Fricke et al., 2006) and therefore cannot support growth when H_2_ is absent (Leahy et al., 2010).

An increase in Proteobacteria, mainly *Succinivibrio* and *Succinimonas* sp., was observed and there was a strong negative correlation between these genera and daily CH4 emissions in the ENS group. This highlights the importance of these bacterial groups in methane mitigation through H_2_ consumption, these bacteria contribute to fumarate reductase activity, increasing reductive reactions in the rumen (Asanuma and Hino, 2000), resulting in less free H_2_ and consequently, lower methane production. Such negative correlations were also observed in other herbivores with lower methane emissions (Pope et al., 2011; Lopes et al., 2015; Danielsson et al., 2017). In addition, in ENS we observed increases in bacteria with reported fumarate reduction capacity, such as *Selenomonas* and *Veillonella* sp. Both were correlated negatively with daily CH4 emissions, suggesting that ENS modulated rumen bacteria for another reductive reaction important in the rumen.

Firmicutes and Bacteroidetes are the most common organisms in rumen microbiota performing essential functions in energy conversion (Granja-Salcedo et al., 2017). In humans, Firmicutes have been correlated with obese populations (Koliada et al., 2017) and genes associated with nutrient transporters, suggesting that Firmicutes is more effective than Bacteroidetes in promoting efficient absorption of calories and subsequent weight gain (Turnbaugh et al., 2006; Krajmalnik-Brown et al., 2012). Thus, the higher abundance of Firmicutes in ENS could be associated to the improved ADG observed in ENS steers.

The reduction in the proportion of ruminal acetate 12 h after supplementation and higher butyrate concentrations by ENS is in line with results from trials of mixed cultures of rumen microbes incubated with nitrate and nitro-compounds (Zhou et al., 2012; Mamvura et al., 2014; Anderson et al., 2016). It may be a compensatory route for the dispensing of reducing equivalents during the inhibition of rumen methanogenesis, shifting electron transfer mainly to more reduced fatty acids such as propionate and butyrate, as this subsequently results in less acetate accumulation (Van Nevel and Demeyer, 1996).

The majority of ruminal propionate production includes species interactions between succinate producers, and succinate to propionate reducer species (Wolin et al., 1997). Even unobserved differences in rumen propionate concentrations between ENS and URS groups, changes in bacterial composition by ENS indicate that the pathway to succinate and propionate production in the rumen were stimulated, once ENS increased succinate and propionate-forming bacteria, such as *Bacteroides, Fibrobacter, Selenomonas, Succinivibrio*, *Veillonella* sp., and were also observed a negative correlation between acetate concentration and Proteobacteria, *Selenomonas* and *Veillonella* sp. Our data showed that ENS tended to increase rumen propionate as did data from Ungerfeld and Kohn (2006) who observed propionogenesis fostering during NO_3_^-^ conversion to NH_3_.

Several studies have observed the toxic effects of nitrite on cellulolytic bacteria populations (Iwamoto et al., 2002; Asanuma et al., 2015) possibly due to the negative effects of both nitrate and nitrite on cellulolytic and xylanolytic activity (Marais et al., 1988). However, cellulolytic bacteria, such as *Ruminococcus*, were not affected by ENS; in contrast, the abundance of *Fibrobacter* increased in the rumen of ENS steers. This may be correlated with the adaptation capacity of *Ruminococcus albus* and *Fibrobacter succinogenes* as shown by Zhou et al. (2012) *in vitro* ruminal cultures with nitrate additions. Similar to our findings, Zhao et al. (2015) found that the main cellulolytic bacteria in the rumen were stimulated by nitrate supplementation in steers.

An important reduction in Verrucomicrobia abundance was observed in ENS groups, which may indicate a reduction in H_2_ rumen pressure by ENS. Species belonging to this phylum have been identified as possible sensitive indicators of H_2_ partial pressures in the rumen, as they are negatively correlated with ruminal H_2_ accumulation during methane inhibition (Martinez-Fernandez et al., 2016). However, the role of this low abundant phylum within the rumen microbiome remains unclear due to limited information about the species within the phylum (Deusch et al., 2017).

Ammonia concentrations in ENS groups were lower than in URS groups; it is possible that EN enables the slow release of ammonia into the rumen, thus improving its use by rumen bacteria, mainly cellulolytic bacteria, to synthesize amino acids required for their growth. This is supported by the negative correlation observed between ammonia concentrations and *Fibrobacter* and *Ruminococcus* sp. Lee et al. (2015a) and Hulshof et al. (2012) also found that rumen ammonia concentration decreased with nitrate supplementation, even with the use of iso-nitrogenous diets, in ruminants. The higher ammonia concentrations observed in URS 6 h after supplementation might be because it takes a longer time for nitrates to be reduced to ammonia compared with the immediate conversion of urea to ammonia in the rumen (Lee et al., 2015a).

Few *in vivo* studies have evaluated bacterial diversity in ruminants supplemented with nitrate, and until now, most of the information about ruminal nitrate and nitrite-reducing bacteria was based on *in vitro* studies. An increase in the abundance of *Veillonella* and *Selenomonas* in ENS was expected, once these bacteria were recognized as nitrate and nitrite-reducing microorganism in the rumen (Iwamoto et al., 2002; Asanuma et al., 2015). However, the observed positive correlation between ammonia concentrations and *Bacteroides*, *Prevotella*, *Kandleria*, *Duganella*, and *Succinivibrio* in ENS groups suggests a possible role of these groups in nitrate and nitrite reduction in the rumen.

Interestingly, the stimulation of some lactate-producer bacteria was observed in ENS (e.g., *Kandleria* and *Lactobacillus*). *Kandleria* is a lactate producing bacteria that has been associated with low-CH_4_ rumino-types in sheep (Kittelmann et al., 2014), explaining the negative correlation between this genus and CH_4_ emissions expresses as g/day and as g/kg of supplement DMI in ENS. The promotion of *Lactobacillus* growth by ENS could be correlated with the nitrate-reducing capacity of some *Lactobacillus* species, which suggests that they may use nitrates as electron acceptors (Brooijmans et al., 2009; Tiso and Schechter, 2015).

The higher rumen pH observed in ENS groups after 6 h of supplementation corresponds with the results of previous studies testing the effects of nitrate use on ruminal fermentation (Zhou et al., 2012; Asanuma et al., 2015; Lee et al., 2015a) and could explain the lower total VFA concentrations observed after 6 h in the ENS group. Lower total VFA concentrations after nitrate addition have been previously observed (Van Zijderveld et al., 2010; Zhou et al., 2012; Mamvura et al., 2014) and may be due to the selective consumption of individual VFA by nitrate-reducing bacteria (Zhou et al., 2012). For example, in our study ENS resulted in a lower proportion of the *iso*-valerate rumen.

In conclusion, EN is a feed additive that persistently affects enteric methane emissions in grazing steers, decreasing *Methanobrevibacter* abundance in the rumen. In addition, ENS may promote fumarate-reducer and lactate-producer bacteria, reducing acetate production during rumen fermentation.

## Data Availability

The sequences of the microbial diversity have been deposited in GenBank under accession number PRJNA506421.

## Author Contributions

RF, RCA, FDR, AB, and GS conceived and designed the experiments. RF performed the experiments. RF, AB, and YG-S performed the laboratorial analysis. LK performed the bioinformatical analysis. YG-S performed the statistical analysis and wrote the manuscript. YG-S, RCA, FDR, GS, and TB reviewed the final manuscript.

## Conflict of Interest Statement

RCA is R&D Manager of GRASP Ind. & Com. LTDA (Curitiba, Brazil), the manufacturer of the encapsulated product evaluated in this study. The remaining authors declare that the research was conducted in the absence of any commercial or financial relationships that could be construed as a potential conflict of interest.

## Footnotes

1 https://github.com/vsbuffalo/scythe
