# Corrigendum: Long-Term Encapsulated Nitrate Supplementation Modulates Rumen Microbial Diversity and Rumen Fermentation to Reduce Methane Emission in Grazing Steers

**DOI:** 10.3389/fmicb.2019.01732

**Published:** 2019-07-29

**Authors:** Yury Tatiana Granja-Salcedo, Rodolfo Maciel Fernandes, Rafael Canonenco de Araujo, Luciano Takeshi Kishi, Telma Teresinha Berchielli, Flávio Dutra de Resende, Alexandre Berndt, Gustavo Rezende Siqueira

**Affiliations:** ^1^Department of Animal Science, Faculdade de Ciências Agrárias e Veterinárias, UNESP – Universidade Estadual Paulista, Jaboticabal, Brazil; ^2^Department of Animal Science, Agência Paulista de Tecnologia dos Agronegócios, Colina, Brazil; ^3^GRASP Ind. & Com. LTDA, Curitiba, Brazil; ^4^Department of Technology, Faculdade de Ciências Agrárias e Veterinárias, UNESP – Universidade Estadual Paulista, Jaboticabal, Brazil; ^5^INCT/CA – UFV, Department of Animal Science, Viçosa, Brazil; ^6^Embrapa Southeast Livestock, São Carlos, Brazil

**Keywords:** archaea diversity, beef cattle, enteric methane emission, nitrate, rumen bacteria diversity, volatile fatty acids

In the original article, we neglected to include the funder “São Paulo Research Foundation (FAPESP, Brazil), 2019/06599-8.”

A correction has therefore been made to the **Funding** statement:

The authors thank GRASP Ind. & Com. LTDA (Curitiba, Brazil) for funding this study and also the São Paulo Research Foundation (FAPESP, Brazil), under grant numbers 2017/02034-0 and 2019/06599-8, for partially supporting this research. The funder was involved in the study design, however, was not involved in collection, analysis, interpretation of data, the writing of this article or the decision to submit it for publication.

The authors apologize for this error and state that this does not change the scientific conclusions of the article in any way. The original article has been updated.

