# Supplementary material for: Long-Term Encapsulated Nitrate Supplementation Modulates Rumen Microbial Diversity and Rumen Fermentation to Reduce Methane Emission in Grazing Steers
Source: Front Microbiol. 2019 Mar 29;10:614. doi: 10.3389/fmicb.2019.00614 (PMC6449429; doi:10.3389/fmicb.2019.00614)
Supplement: Supplementary file 1 [file Table_1.pdf]

Long-term encapsulated nitrate supplementation modulates rumen microbial diversity and rumen fermentation to reduce methane emission in grazing steers

Yury Tatiana Granja-Salcedo[1*], Rodolfo Maciel Fernandes[1,2], Rafael Canonenco de Araujo[3], Luciano Takeshi Kishi[4], Telma Teresinha Berchielli[1,5], Flávio Dutra de Resende[1,2], Alexandre Berndt [6], Gustavo Rezende Siqueira[1,2]

[1] Department of Animal Science, Faculdade de Ciências Agrárias e Veterinárias (FCAV), Univ Estadual Paulista–UNESP, Jaboticabal, SP, Brazil.

[2] Department of Animal Science, Agência Paulista de Tecnologia dos Agronegócios (APTA), Colina, SP, Brazil.

[3] GRASP Ind. & Com. LTDA, Curitiba, PR, Brazil

[4] Department of Technology, Faculdade de Ciências Agrárias e Veterinárias (FCAV), Univ Estadual Paulista–UNESP, Jaboticabal, SP, Brazil.

[5] INCT/CA–UFV-Department of Animal Science, Viçosa, MG, Brazil.

[6] Embrapa Southeast Livestock, São Carlos, SP, Brazil.

***Correspondence:** Dr. Yury T. Granja-Salcedo. yurygranjasalcedo@gmail.com

Supplemental material

Table S1. Ingredients and chemical composition of supplements offered and forage nutritional value during each period.

	Dry season (135 d)		Rainy season (168 d)		Finished (104 d)	
Ingredient, g/kg DM	**UR**	**EN**	**UR**	**EN**	**UR**	**EN**
Soybean meal	198.9	198.2	0.0	0.0	69.8	69.3
Ground corn	654.9	652.4	814.0	809.3	876.4	870.0
Encapsulated Nitrate	0.0	99.2	0.0	139.0	0.0	48.4
Urea	34.2	0.0	47.9	0.0	14.8	0.0
Calcareous	61.7	0.0	86.1	0.0	26.6	0.0
Mineral supplement	50.3	50.2	52.0	51.7	12.4	12.3
Supplement chemical composition, %						
Crude protein	26.0	26.0	21.0	21.0	16.0	16.0
NDT	71.0	71.0	69.0	69.0	79.0	79.0
Forage nutritional value, g/kg DM						
Crude protein	45.6		111.3		97.2	
Ether extract	12.6		14.0		18.2	
Neutral detergent fibre	722.8		658.6		683.5	
Acid detergent fibre	436.0		351.1		349.0	

Table S2. Median and interquartile range of the number of observed operational taxonomic units (OTUs), richness (Chao1 and ACE) and diversity estimators (Shannon Wiener and Simpson) by rumen bacteria and archaea population in grazing Nellore Steers after thirteen months of supplementation with Urea (URS) or encapsulated nitrate (ENS).

	URS	ENS	p-value
**Bacteria**			
Total OTUs	2030±80	2060±317	0.9048
Chao-1	2175±119	2199±273	0.8923
ACE	2190±125	2191±227	0.6342
Shannon	5.893±0.20	5.906±0.48	0.9137
Simpson	0.966±0.02	0.968±0.01	0.8906
**Archaea**			
Total OTUs	457.18±51	439.32±27	0.7381
Chao-1	498.47±47	471.64±34	0.7925
ACE	489.46±27	490.32±27	0.9183
Shannon	1.587±0.17	1.479±0.41	0.3874
Simpson	0.959±0.01	0.951±0.01	0.5962

Table S3. Median and interquartile range of the bacterial abundance at phylum level (a) and Euryarchaeota:Bacteria ratio (b) in grazing Nellore Steers after thirteen months of supplementation with Urea (URS) or encapsulated nitrate (ENS).

	Supplement		p-value
**Bacterial phylum**	**URS**	**ENS**	
Actinobacteria	0.145±0.02	1.494±0.24	0.0447
Bacteroidetes	41.331±11.61	39.339±6.65	0.7302
Cyanobacteria	0.238±0.40	0.342±0.22	0.5556
Fibrobacteres	2.181±1.06	3.283±2.22	0.0412
Firmicutes	23.005±6.13	30.498±8.31	0.0017
Lentisphaerae	0.436±0.49	0.503±0.51	0.1197
Proteobacteria	2.581±1.26	7.985±1.90	0.0392
Spirochaetes	2.335±1.25	1.149±1.04	0.1256
SR1	0.753±0.42	0.836±0.35	0.6124
Synergistetes	0.079±0.05	0.098±0.06	0.1933
Tenericutes	1.163±0.21	0.639±0.17	0.6382
TM7	0.853±0.31	0.793±0.24	0.6904
Verrucomicrobia	6.985±0.46	1.126±0.82	0.0031
WPS.2	0.409±0.14	0.283±0.11	0.7038
Unassigned	16.303±3.55	11.581±3.48	0.0101

Differences were considered significant at $p < 0.05$ using the Wilcoxon test.

Table S4. Spearman correlations (r) and P-values (p) among microbial groups and methane emissions in grazing Nellore Steers after thirteen months of supplementation with Urea (URS) or encapsulated nitrate (ENS).

		DMI, kg/d				CH4, gr/d				CH4, gr/kg tDMI				CH4, gr/kg sDMI				CH4, gr/kg fDMI				Rumen pH			
		URS		ENS		URS		ENS		URS		ENS		URS		ENS		URS		ENS		URS		ENS	
**Amplicon**	**Microbial taxa**	**r**	**p**	**r**	**p**	**r**	**p**	**r**	**p**	**r**	**p**	**r**	**p**	**r**	**p**	**r**	**p**	**r**	**p**	**r**	**p**	**r**	**p**	**r**	**p**
Archaea	Euryarchaeota							0.64	0.07							0.70	0.05					-0.77	0.04		
	Methanobacterium																								
	Methanobrevibacter																	-0.77	0.04			-0.66	0.08		
	Methanomassiliicoccus					-0.89	0.02			-0.89	0.02			-0.89	0.02	0.66	0.07								
Bacteria	Actinobacteria	0.99	0.01															-0.81	0.03						
	Bacteroidetes			0.87	0.02																				
	Cyanobacteria			0.75	0.04					-0.67	0.08			-0.67	0.08										
	Fibrobacteres	0.99	0.01					0.81	0.02									-0.81	0.03						
	Firmicutes																								
	Proteobacteria			-0.70	0.05			-0.70	0.05									-0.67	0.08	0.64	0.07				
	Spirochaetes	0.90	0.01									0.67	0.07					-0.90	0.08						
	Tenericutes					0.70	0.05			0.70	0.05			0.70	0.05										
	Verrucomicrobia																								
	BF ratio																								
	BactBlautia																								
	Bacteroides					-0.77	0.04			-0.77	0.04	-0.77	0.04	-0.77	0.04	-0.89	0.02								
	Barnesiella	-0.77	0.04	0.71	0.05			0.89	0.02																
	Clostridium_III									0.83	0.03			0.83	0.03										
	Clostridium_IV					-0.77	0.04			-0.77	0.04			-0.77	0.04	0.77	0.04					-0.66	0.08		
	Clostridium_XlVa			-0.77	0.04			-0.77	0.04	0.75	0.04			0.75	0.04			0.70	0.05	0.66	0.07				
	Duganella															-0.77	0.04								
	Eubacterium									0.83	0.03			0.83	0.03	0.77	0.04	0.66	0.08						
	Fibrobacter																					-0.89	0.02		
	Kandleria	0.83	0.03					-0.83	0.03							-0.83	0.03								
	Lactobacillus			-0.70	0.05																	-0.78	0.04		
	Mitsuokella																								
	Mogibacterium									0.66	0.08			0.66	0.08										
	Olsenella																					-0.89	0.02		
	Paraprevotella									0.77	0.04	-0.89	0.02	0.77	0.04					-0.83	0.03				
	Prevotella															-0.94	0.01								
	Pseudobutyrivibrio	-0.71	0.05															0.83	0.03						
	Roseburia							-0.83	0.03																
	Ruminococcus							0.71	0.05							0.77	0.04								
	Selenomonas							-0.94	0.01															-0.66	0.08
	Sphaerochaeta			0.71	0.05			0.89	0.01																
	Succinimonas	-0.71	0.05			-0.83	0.03															-0.71	0.05		
	Succinivibrio							-0.71	0.05							-0.77	0.04								
	Treponema																					-0.89	0.02		
	Veillonella							-0.83	0.03																

CH4 = methane; sDMI = supplement dry matter intake; fDMI = forage dry matter intake; tDMI = total dry matter intake. Only significant P < 0.10 correlations are shown.

Table S4. Spearman correlations (r) and P-values (p) among microbial groups and rumen fermentation parameters in grazing Nellore Steers after thirteen months of supplementation with Urea (URS) or encapsulated nitrate (ENS).

		**Rumen ammonia**				**Acetate, %**				**Propionate, %**				**Isobutyrate. %**				**Butyrate, %**				**Isovalerate, %**			
		**URS**		**ENS**		**URS**		**ENS**		**URS**		**ENS**		**URS**		**ENS**		**URS**		**ENS**		**URS**		**ENS**	
**Amplicon**	**Microbial taxa**	**r**	**p**	**r**	**p**	**r**	**p**	**r**	**p**	**r**	**p**	**r**	**p**	**r**	**p**	**r**	**p**	**r**	**p**	**r**	**p**	**r**	**p**	**r**	**p**
Archaea	Euryarchaeota	-1.00	0.01	-0.93	0.01							-0.75	0.05	-0.94	0.01							-0.77	0.04		
	Methanobacterium			-0.71	0.05	0.77	0.04			-0.89	0.02	-0.77	0.04							0.66	0.07	0.66	0.07		
	Methanobrevibacter	-0.94	0.01	-0.89	0.02							-0.71	0.05	-0.89	0.02							-0.71	0.05		
	Methanomassiliicoccus			-0.83	0.03							-0.77	0.04					0.77	0.04						
Bacteria	Actinobacteria											0.64	0.07												
	Bacteroidetes							0.81	0.03															-0.87	0.02
	Cyanobacteria			0.67	0.07													0.78	0.04					-0.84	0.03
	Fibrobacteres			-0.75	0.05							-0.93	0.01												
	Firmicutes													0.70	0.05			-0.81	0.03						
	Proteobacteria							-0.99	0.01											-0.84	0.03				
	Spirochaetes			-0.84	0.03											-0.93	0.01								
	Tenericutes											-0.78	0.04												
	Verrucomicrobia			-0.90	0.01	0.67	0.07			-0.75	0.04					-0.87	0.02								
	BF ratio									-0.64	0.08													-0.77	0.04
	BactBlautia	-0.77	0.04	-0.89	0.02									-0.83	0.03										
	Bacteroides			0.83	0.03	-0.71	0.05											0.66	0.08						
	Barnesiella	0.71	0.05			-0.66	0.08	0.89	0.02					0.77	0.04					0.83	0.03				
	Clostridium_III			-0.94	0.01																				
	Clostridium_IV			-0.94	0.01							-0.66	0.07			-0.71	0.05	0.89	0.02			-0.71	0.05		
	Clostridium_XlVa							-0.83	0.03											-0.71	0.05				
	Duganella	0.82	0.03	0.94	0.01							0.66	0.07	0.70	0.05	0.71	0.05								
	Eubacterium			-0.94	0.01							-0.66	0.07			-0.71	0.05								
	Fibrobacter	-0.77	0.04	-0.71	0.05					0.71	0.05	-0.77	0.04	-0.66	0.08					0.66	0.07	-1.00	0.01		
	Kandleria			0.89	0.02	0.77	0.04																		
	Lactobacillus									0.64	0.08					-0.64	0.07								
	Mitsuokella					0.94	0.01			-0.83	0.03														
	Mogibacterium			-0.71	0.05							-0.77	0.04	-0.66	0.08					0.66	0.07				
	Olsenella	-0.77	0.04	-0.83	0.03					0.71	0.05			-0.66	0.08							-1.00	0.01		
	Paraprevotella							0.89	0.02											0.66	0.07			-0.66	0.07
	Prevotella			0.83	0.03							0.71	0.05												
	Pseudobutyrivibrio																								
	Roseburia					0.71	0.05	-0.83	0.03											-0.71	0.05				
	Ruminococcus			-0.94	0.01					0.77	0.04	-0.77	0.04												
	Selenomonas					0.83	0.03	-0.77	0.04	-0.66	0.08									-0.77	0.04				
	Sphaerochaeta							0.89	0.02											0.83	0.03				
	Succinimonas					-0.66	0.08															-0.66	0.07		
	Succinivibrio			0.94	0.01							0.77	0.04												
	Treponema			-0.77	0.04					0.66	0.08					-0.66	0.07					-0.83	0.03		
	Veillonella					0.83	0.03	-0.83	0.03	-0.71	0.05									-0.71	0.05				

Table S4. Spearman correlations (r) and P-values (p) among microbial groups and methane emissions in grazing Nellore Steers after thirteen months of supplementation with Urea (URS) or encapsulated nitrate (ENS).

		Valerate, %				Total VFA, mmol/L			
		URS		ENS		URS		ENS	
**Amplicon**	**Microbial taxa**	**r**	**p**			**r**	**p**	**r**	**p**
Archaea	Euryarchaeota					0.89	0.02		
	Methanobacterium	-0.77	0.04						
	Methanobrevibacter					0.77	0.04		
	Methanomassiliicoccus								
Bacteria	Actinobacteria							0.93	0.01
	Bacteroidetes			-0.70	0.05			0.64	0.07
	Cyanobacteria								
	Fibrobacteres			-0.81	0.03				
	Firmicutes					-0.64	0.07	-0.67	0.07
	Proteobacteria			0.78	0.04				
	Spirochaetes								
	Tenericutes			-0.67	0.07				
	Verrucomicrobia	-0.67	0.07						
	BF ratio							0.83	0.03
	BactBlautia					0.66	0.07		
	Bacteroides	0.71	0.05						
	Barnesiella	0.66	0.07	-0.94	0.01				
	Clostridium_III					0.71	0.05		
	Clostridium_IV								
	Clostridium_XlVa			0.89	0.02				
	Duganella								
	Eubacterium								
	Fibrobacter					0.77	0.04		
	Kandleria	-0.77	0.04	0.66	0.07				
	Lactobacillus								
	Mitsuokella	-0.94	0.01					0.89	0.02
	Mogibacterium					0.83	0.03		
	Olsenella					0.77	0.04		
	Paraprevotella								
	Prevotella								
	Pseudobutyrivibrio							-0.94	0.01
	Roseburia	-0.71	0.05	0.77	0.04				
	Ruminococcus								
	Selenomonas	-0.83	0.03	0.89	0.02				
	Sphaerochaeta			-0.94	0.01				
	Succinimonas	0.66	0.07					-0.94	0.01
	Succinivibrio								
	Treponema								
	Veillonella	-0.83	0.03	0.77	0.04				

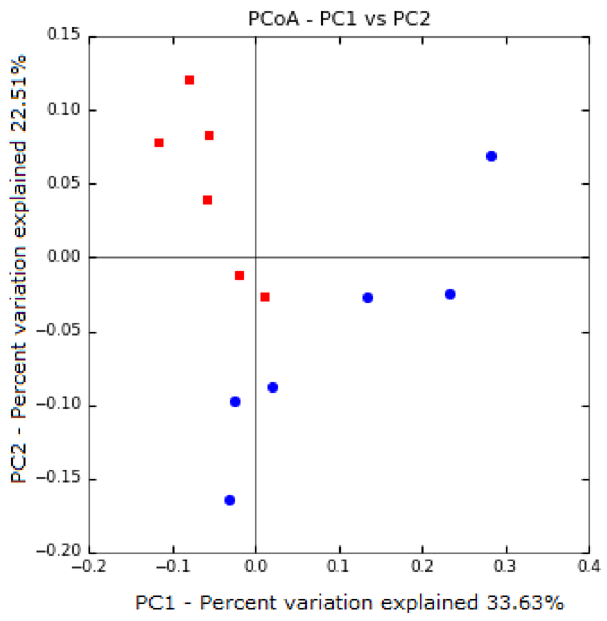

Figure S.1. Weighted Unifrac diversity principal coordinate analysis (PCoA) to explore dissimilarities in microbial composition among grazing Nellore Steers after a long time (thirteen months) of supplementation with encapsulated nitrate (blue circle) or urea (red square). Unifrac test = 0.061.
